# *AcMYB176*-Regulated *AcCHS5* Enhances Salt Tolerance in *Areca catechu* by Modulating Flavonoid Biosynthesis and Reactive Oxygen Species Scavenging

**DOI:** 10.3390/ijms26073216

**Published:** 2025-03-30

**Authors:** Yiqi Jiang, Noor Muhammad Khan, Akhtar Ali, Guangzhen Zhou, Yue Zhou, Panjing Li, Yinglang Wan

**Affiliations:** 1 Hainan Key Laboratory for Sustainable Utilization of Tropical Bioresources, School of Tropical Agriculture and Forestry, Hainan University, Haikou 570228, China; jyq217317@163.com (Y.J.); noormaghmoom@gmail.com (N.M.K.); anaakhtar631@gmail.com (A.A.); panjingli2025@163.com (P.L.); 2 The Ministry of Education Key Laboratory for Ecology of Tropical Islands, Key Laboratory of Tropical Animal and Plant Ecology of Hainan Province, College of Life Sciences, Hainan Normal University, Haikou 571158, China; gzzhou@hainanu.edu.cn (G.Z.); amasakikiha@163.com (Y.Z.)

**Keywords:** *Areca catechu*, *CHS* gene family, salt stress, *AcCHS5*, flavonoid biosynthesis

## Abstract

High-salinity stress induces severe oxidative damage in plants, leading to growth inhibition through cellular redox imbalance. Chalcone synthase (CHS), a pivotal enzyme in the flavonoid biosynthesis pathway, plays critical roles in plant stress adaptation. However, the molecular mechanisms underlying CHS-mediated salt tolerance remain uncharacterized in *Areca catechu* L., a tropical crop of high economic and ecological significance. Here, we systematically identified the *CHS* gene family in *A. catechu* and revealed tissue-specific and salt-stress-responsive expression patterns, with *AcCHS5* exhibiting the most pronounced induction under salinity. Transgenic *Arabidopsis* overexpressing *AcCHS5* displayed enhanced salt tolerance compared to wild-type plants, characterized by elevated activities of antioxidant enzymes: superoxide dismutase (SOD), catalase (CAT), and peroxidase (POD), increased flavonoid accumulation, and reduced reactive oxygen species (ROS) accumulation. Furthermore, we identified the transcription factor *AcMYB176* as a direct activator of *AcCHS5* through binding to its promoter. Our findings demonstrate that the *AcMYB176-AcCHS5* regulatory module enhances salt tolerance by orchestrating flavonoid biosynthesis and ROS scavenging. This study provides functional evidence of CHS-mediated salt adaptation in *A. catechu* and highlights its potential for improving stress resilience in tropical crops.

## 1. Introduction

Salt stress is one of the most common abiotic stresses in nature, and soil salinization can constrain plant growth and development. When the soil salt concentration is too high, the water potential outside plant cells decreases, impairing the root system’s ability to absorb water normally [1]. Additionally, excessive sodium (Na^+^) and chloride (Cl^−^) ions enter the cells, interfering with the plant’s absorption of essential nutrients such as potassium (K^+^) and calcium (Ca^2^^+^), leading to ionic toxicity [2]. Salt stress can further induce oxidative stress, resulting in the accumulation of reactive oxygen species (ROS), which affects plant metabolism and biochemical reactions [2]. To cope with salt stress, plants have evolved various adaptive mechanisms, such as using ion transport proteins to exclude excessive sodium ions or compartmentalize them in vacuoles [3]. Additionally, plants activate antioxidant enzyme systems, including superoxide dismutase (SOD), peroxidase (POD), and catalase (CAT), and synthesize osmoprotectants and antioxidants like proline (Pro), glycinebetaine, and flavonoid compounds [4]. These mechanisms help to scavenge reactive ROS generated by salt stress, reduce cellular damage, and enhance plant tolerance to salinity.

Chalcone synthase (CHS), a pivotal rate-limiting enzyme in the flavonoid biosynthesis pathway, governs the formation of chalcone—the foundational carbon skeleton for diverse flavonoid derivatives. In the phenylpropanoid pathway, CHS catalyzes the condensation of p-coumaroyl-CoA and malonyl-CoA to generate naringenin chalcone, which serves as the precursor for subsequent enzymatic modifications. Downstream metabolic reactions convert chalcone into flavanols, flavonols, anthocyanins, and other specialized metabolites through hydroxylation, glycosylation, and isomerization processes [5]. Beyond its biosynthetic role, CHS-mediated flavonoid production is integral to plant stress adaptation. These compounds function as antioxidants to neutralize reactive ROS under abiotic (e.g., salinity [6], drought [7], and chilling stress [8]) and biotic challenges (e.g., UV exposure and pathogen invasion [9]). Emerging evidence highlights CHS as a molecular determinant of stress resilience. For instance, transgenic tobacco overexpressing *NtCHS1* exhibits elevated rutin accumulation under salt stress, which correlates with enhanced ROS scavenging capacity, reduced hydrogen peroxide (H_2_O_2_) and superoxide anion (O_2_·^−^) levels, and improved survival rates [10]. Similarly, drought-stressed *CpCHS1*-overexpressing tobacco (derived from *Prunus avium* L.) demonstrates superior biomass retention, concomitant with upregulated antioxidant enzymes and Pro biosynthesis [11]. These findings underscore the dual role of CHS in fortifying redox homeostasis and osmotic adjustment during stress adaptation.

MYB transcription factors, a class of ubiquitously present regulatory proteins in plants, have been demonstrated to modulate flavonoid metabolism primarily by interacting with structural genes in the flavonoid biosynthetic pathway [12]. These structural genes typically encode key enzymes involved in flavonoid biosynthesis, including CHS, flavanone 3-hydroxylase (F3H), and flavonol synthase (FLS). Through this regulatory network, MYB transcription factors coordinate diverse secondary metabolic processes, thereby exerting precise control over the flavonol branch pathway [13]. Subsequent studies have cloned *MYB* genes in diverse plant species including arabidopsis (*Arabidopsis thaliana* L.) [14], potato (*Solanum tuberosum* L.) [15], nectarine (*Prunus persica* (L.) Batsch) [16], and ginkgo (*Ginkgo biloba* L.) [17], with functional characterization demonstrating their regulatory roles in the biosynthesis of flavonoids and other secondary metabolites. In soybean (*Glycine max* (L.) Merr.), *GmMYB12B2* demonstrates the capacity to significantly upregulate multiple key enzyme-encoding genes within the flavonoid biosynthetic pathway, consequently enhancing flavonoid biosynthesis [18].

*Areca catechu* L., a tree-like tropical plant of the family Arecaceae and genus Areca, is globally recognized as one of the quintessential tropical cash crops. Originating from Malaysia, it is primarily cultivated in regions such as Hainan, Guangxi, Guangdong, Yunnan, and Taiwan in China [19]. Furthermore, areca, a masticatory commodity in China and one of the nation’s “Four Great Southern Medicinal Herbs”, contains diverse bioactive constituents including lipids (9.15%), alkaloids (5%), tannins (15%), and other phytochemicals, endowing it with significant industrial utility [20]. The phytochemical profiling of betel nuts has revealed a spectrum of compounds, predominantly alkaloids, flavonoids, tannins, triterpenoids, and fatty acids [21]. Salt stress significantly impairs the growth and development of *A. catechu* [22]. However, the regulatory mechanisms of *CHS* genes in the flavonoid metabolic pathway and their role in the salt tolerance of *A. catechu* remain elusive. Therefore, elucidating the molecular mechanisms underlying *A. catechu*’s response to high-salinity stress holds crucial significance for cultivating salt-tolerant *A. catechu* varieties. This study investigates the *CHS* gene family in *A. catechu*, functional characterization of *CHS* genes, and their upstream regulatory networks, aiming to decipher CHS-mediated flavonoid biosynthesis and salt stress resistance mechanisms. 

## 2. Results

### 2.1. Phylogenetic Analysis of CHS Family

**Figure 1:**
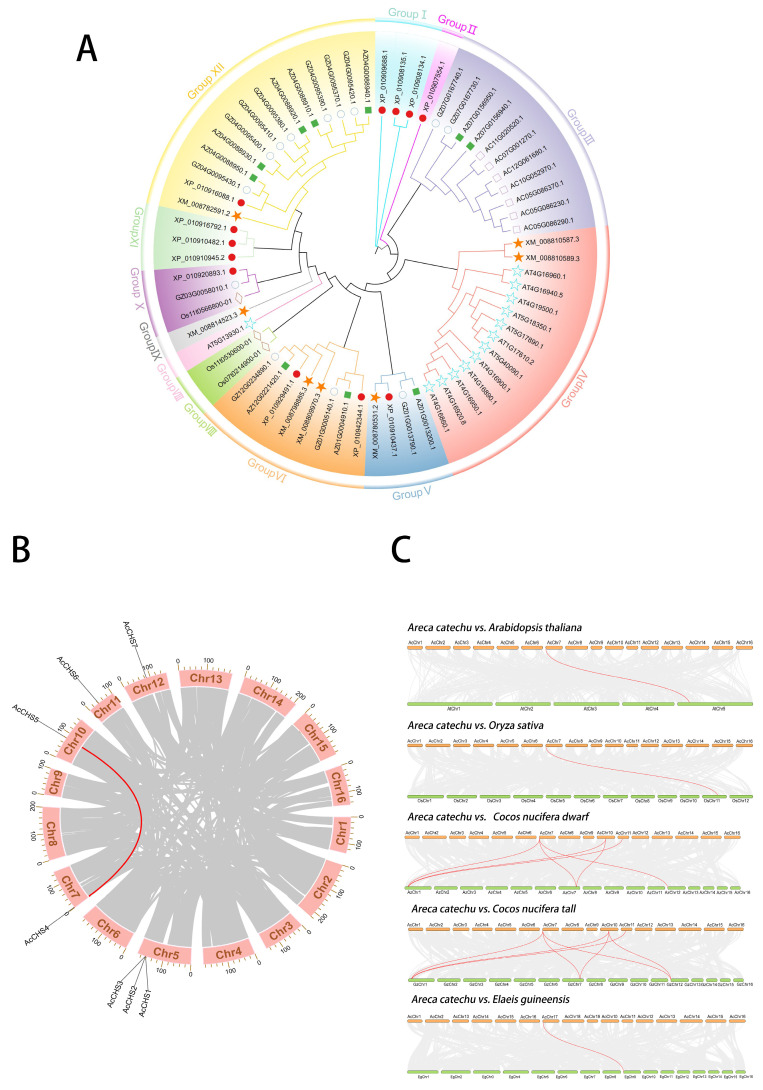
Phylogenetic and syntenic analyses of *CHS* genes in *A. catechu.* (**A**) Unrooted neighbor-joining phylogenetic tree of CHS proteins from *A. catechu* and six representative species (*A. thaliana*, *O. sativa*, * C. nucifera dwarf*, * C. nucifera tall*, * P. dactylifera*, and *E. guineensis*), constructed using MEGA 11. Twelve major clades (I–XII) are color coded. (**B**) Intra-species synteny of *AcCHS* loci (red pentagons) on *A. catechu* chromosomes. Paralogous duplication events are indicated by red connecting lines; gray lines represent collinear blocks. (**C**) Cross-species microsynteny networks between *A. catechu* and five plants. Conserved *CHS* orthologs (red stars) are embedded in macro-syntenic regions (gray ribbons).

To investigate the evolutionary relationships between the *CHS* family genes of *A. catechu* and other plant species, a phylogenetic tree was constructed using the neighbor-joining (NJ) method in MEGA-X software. The analysis included seven species, *A. catechu*, rice (*Oryza sativa* L.)*,* Arabidopsis (*Arabidopsis thaliana* L.), tall coconut (*Cocos nucifera tall* L.), dwarf coconut (*Cocos nucifera dwarf* L.), date palm (*Phoenix dactylifera* L.), and oil palm (*Elaeis guineensis* Jacq.), with 66 gene sequences (Table S1). The phylogenetic tree was divided into 12 distinct clades. The *CHS* genes of *A. catechu* exhibited close clustering with those of *C. nucifera dwarf* and *C. nucifera tall*, likely due to their shared taxonomic classification within the Arecaceae family, conserved structural features, and high sequence homology (Figure 1A).

### 2.2. Chromosomal Localization and Intra- and Interspecific Collinearity Analyses of A. catechu CHS Family

To determine the chromosomal distribution of *CHS* family genes in *A. catechu*, we mapped their genomic loci across all 16 chromosomes using TBtools (version 2.1025). Chromosomal assignments revealed the following patterns: *AcCHS1, AcCHS2,* and *AcCHS3* clustered on chromosome 5, while *AcCHS4*, *AcCHS5*, *AcCHS6*, and *AcCHS7* were distributed on chromosomes 7, 10, 11, and 12, respectively (Figure 1B). An evolutionary trajectory analysis identified a single intragenomic collinear gene pair (*AcCHS5-AcCHS4*) within the *AcCHS* family. Interspecific synteny analyses with representative species (*A. thaliana*, *O. sativa*) and Arecaceae members including (*E. guineensis*, *C. nucifera* dwarf, and *C. nucifera* tall) demonstrated distinct evolutionary conservation patterns. The highest collinearity was observed between *A. catechu* and *C. nucifera tall* (seven collinear pairs), followed by *C. nucifera dwarf* (six pairs). Single collinear pairs were detected between *A. catechu* and *A. thaliana*, *O. sativa*, or *E. guineensis* (Figure 1C). A Ka/Ks ratio analysis of all collinear gene pairs across species consistently yielded values <1 (Table 1), indicating the *CHS* gene was predominantly under purifying selection.

### 2.3. Cis-Acting Element Analysis of Areca CHS Family Members

**Figure 2:**
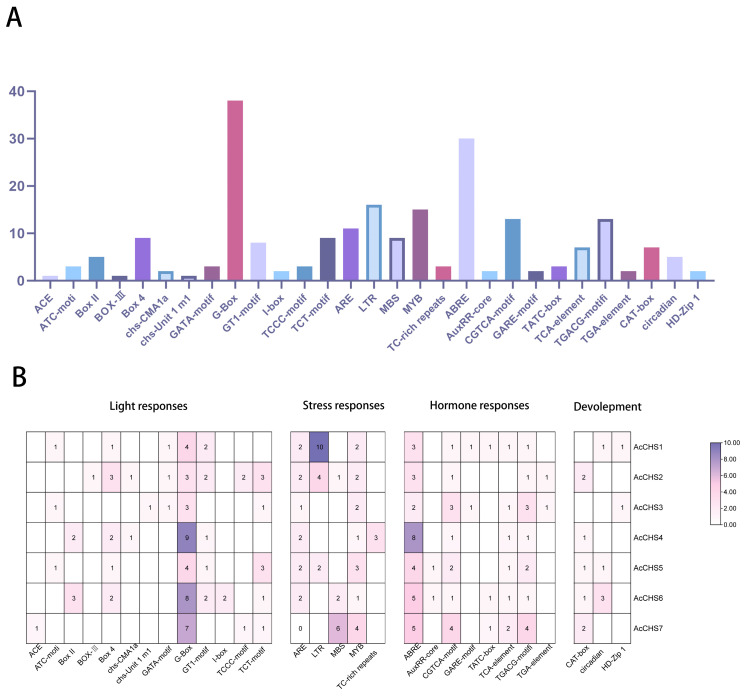
Distribution of *cis*-acting elements in the 2000 bp upstream promoter region of the *AcCHS* gene family. (**A**) Bar chart showing the abundance of regulatory elements across response types. The vertical axis indicates the number of elements, and the horizontal axis labels regulatory element categories (e.g., ACE-core, Box 4). (**B**) Heatmap partitioned into four functional categories: light response, stress response, hormone response, and development. Rows represent regulatory elements, columns correspond to *AcCHS1–AcCHS7* genes, and numerical values in cells indicate the association frequency between genes and elements. Color gradient reflects element abundance (darker hues = higher counts).

A comprehensive analysis of cis-acting elements in the promoter regions of *A. catechu CHS* genes identified 225 functional motifs classified into four categories. Light-responsive elements formed the largest proportion (85 elements, 38%), comprising 13 photoperiod-regulated subtypes. Hormone-responsive elements (72 elements, 32%) included GA-box (gibberellin), AuxRR-core (auxin), and ABRE (abscisic acid) motifs. Stress-related elements (54 elements, 24%) contained drought-responsive MYB sites, hypoxia-activated ARE, and low-temperature-inducible LTR elements. Developmental regulators (14 elements, 6%), predominantly circadian rhythm-associated GATA-box motifs, constituted the smallest group (Figure 2A,B). These findings indicate that the transcriptional regulation of *AcCHS* genes integrates light perception, hormonal cues, stress adaptation, and developmental timing, elucidating their functional diversification in *A. catechu.*

### 2.4. Expression Profiling of A. catechu CHS Genes

**Figure 3:**
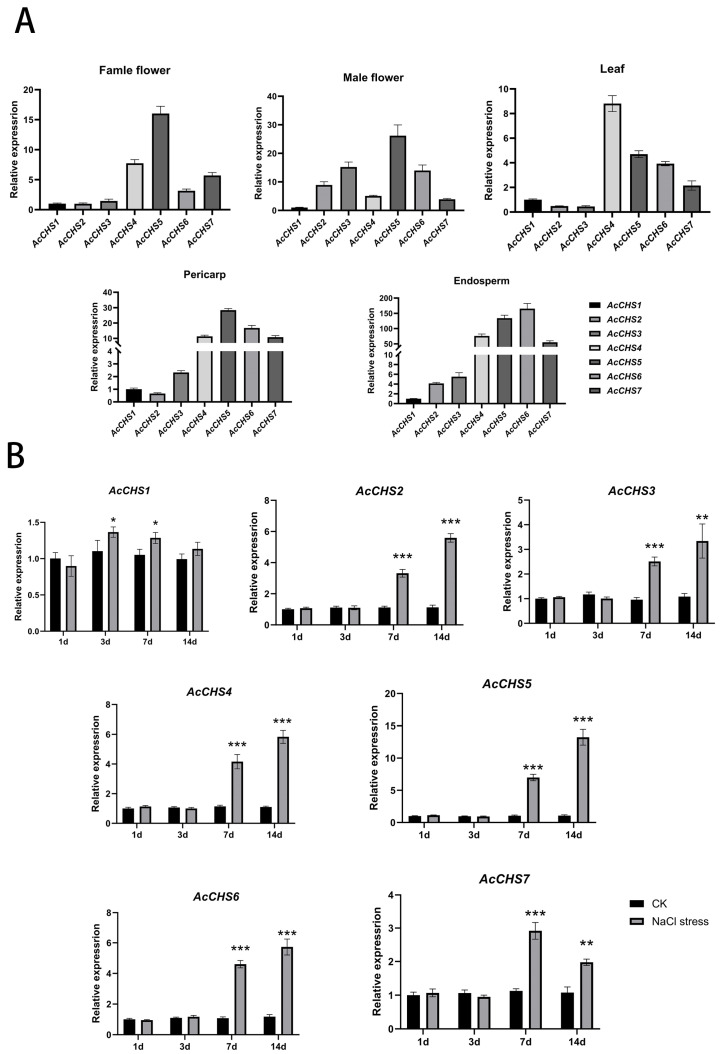
Expression profiles of *AcCHS* gene family members under tissue-specific and salt stress conditions. (**A**) Relative expression levels of *AcCHS1–AcCHS7* in different tissues: female flower, male flower, leaf, pericarp, and endosperm. Bars shown in gray represent individual genes (left to right: *AcCHS1* to *AcCHS7*). (**B**) Time-course expression changes of *AcCHS1–AcCHS7* under control (CK, black bars) and NaCl stress (gray bars) 1, 3, 7, and 14 days post-treatment. Error bars represent mean ± SD from three biological replicates (n = 3). Statistical significance was determined using *t*-test (* *p* < 0.05, ** *p* < 0.01, *** *p* < 0.001).

To delineate the tissue-specific expression patterns of *CHS* genes in *A. catechu*, we performed a quantitative real-time PCR (qRT-PCR) analysis of five tissues: leaf, female flower, male flower, pericarp, and endosperm (Figure 3A). A striking expression hierarchy was observed among *AcCHS* paralogs. *AcCHS5* dominated in reproductive and fruit tissues, displaying 16.0-, 26.2-, and 28.4-fold higher expression levels than *AcCHS1* in female flowers, male flowers, and pericarp, respectively. *AcCHS4* showed leaf-specific predominance with an 8.8-fold increase relative to *AcCHS1*. Notably, *AcCHS6* exhibited high expression in endosperm tissue, exceeding *AcCHS1* expression 165.3-fold, while *AcCHS5* ranked second in this tissue (134.0-fold). In contrast, *AcCHS1*, *AcCHS2*, and *AcCHS3* maintained constitutively low expression levels across all tissues. These results demonstrate the functional diversification of *AcCHS* genes in organ-specific development and metabolic specialization in *A. catechu*.

### 2.5. Expression Patterns Under Salt Stress

To investigate the expression patterns of *CHS* genes under high salinity levels, we quantified the transcriptional levels of *AcCHS1-7* in *A. catechu* leaves using qRT-PCR (Figure 3B). The temporal analysis revealed divergent expression profiles: *AcCHS1* showed no significant changes throughout the salt treatment, while other genes exhibited progressive upregulation. *AcCHS2* expression increased 3.0 fold compared to the control on Day 7 and peaked at 5.0 fold on Day 14. Similarly, *AcCHS3* rose from 2.0 fold to 2.8 fold, and *AcCHS4* escalated from 3.6 fold to 5.2 fold during the same period. *AcCHS5* demonstrated the most pronounced induction, surging from 6.6 fold on Day 7 to 12.3 fold on Day 14—the highest magnitude among all the homologs. *AcCHS6* exhibited moderate upregulation (4.2 fold to 4.8 fold). In contrast, *AcCHS7* reached its maximum expression (2.5 fold) on Day 7 but declined to 1.8 fold by Day 14. The exceptional salt-responsive activation of *AcCHS5*, characterized by its sustained and dominant expression, warranted its selection for subsequent functional studies.

### 2.6. Identification of Transgenic A. thaliana

**Figure 4:**
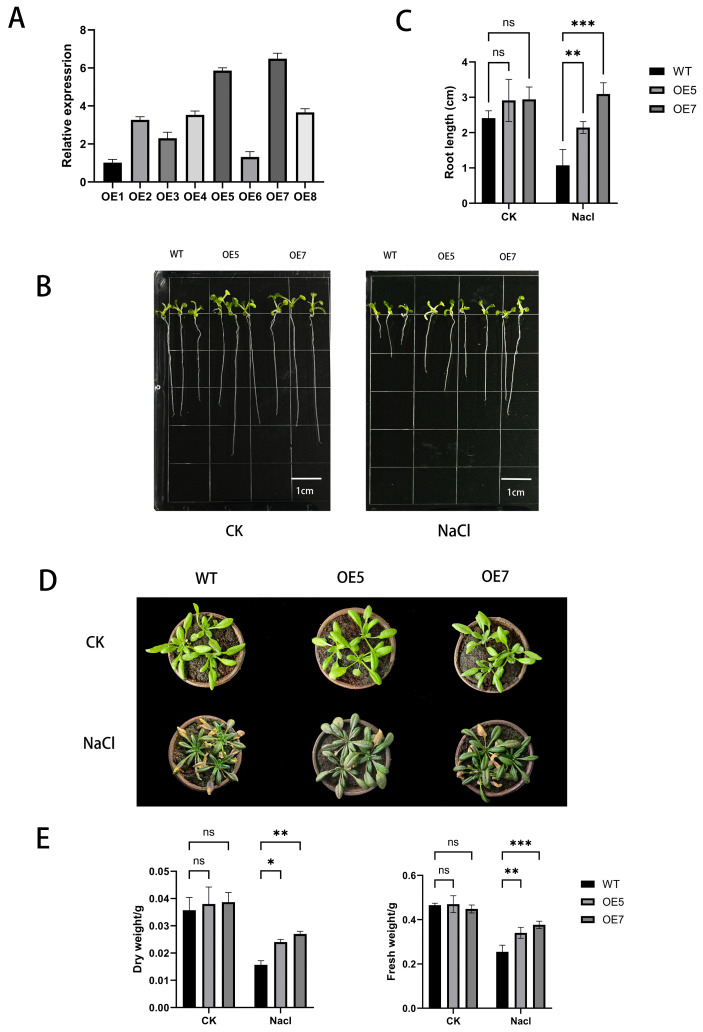
Phenotypic and physiological characterization of *AcCHS5*-OE lines under control and NaCl stress conditions. (**A**) The relative expression of *AcCHS5* in different *A. thaliana* lines. (**B**) Seedling phenotypes of WT, OE5, and OE7 under control (CK, left) and NaCl stress (right). Scale bar = 1 cm. (**C**) Root length measurements of WT (black), OE5 (light gray), and OE7 (dark gray). (**D**) Mature plant phenotypes under CK (upper panel) and NaCl treatment (lower panel). (**E**) Dry weight (left) and fresh weight (right) of plants. Error bars represent mean ± SD from three biological replicates (n = 3). Statistical significance was determined using two-way ANOVA (ns, * *p* < 0.05, ** *p* < 0.01, *** *p* < 0.001).

Through *Agrobacterium tumefaciens*-mediated floral dip transformation, we generated eight independent *AcCHS5*-overexpressing (OE) transgenic lines in *A. thaliana*. Three randomly selected T3-generation single-copy homozygous lines per construct were subjected to transcript-level validation. The total RNA extracted from leaves was reverse-transcribed into cDNA for qRT-PCR analysis. Lines OE5 and OE7 exhibited the highest *AcCHS5* expression levels and were therefore selected for subsequent phenotypic and molecular analyses (Figure 4A).

### 2.7. Phenotypic Evaluation of A. thaliana Under Salt Stress

To assess the salt tolerance conferred by *AcCHS5*, wild-type (WT) and *AcCHS5*-OE-line *A. thaliana* seedlings were subjected to hydroponic culture in standard ½ strength Murashige and Skoog (MS) medium or ½ MS medium containing 200 mmol L^−^^1^ NaCl. Under control conditions, the root lengths of the WT and OE lines showed no significant differences. Strikingly, under 200 mmol L^−^^1^ NaCl stress, the *AcCHS5*-OE lines exhibited a significantly longer primary root length compared to the WT plants (Figure 4B,C). Soil-based salt stress assays revealed distinct morphological responses between the genotypes. While both lines showed similar growth phenotypes under non-stress conditions, the NaCl treatment (200 mmol L^−^^1^) induced characteristic stress symptoms, including leaf chlorosis, necrosis, and curling, in all plants. However, the WT plants showed exacerbated wilting and chlorosis relative to the *AcCHS5*-OE lines (Figure 4D). Biomass quantification demonstrated that the *AcCHS5*-OE lines maintained a significantly higher fresh weight and dry weight relative to the WT lines under salt stress (Figure 4E). Collectively, these data demonstrate that *AcCHS5* overexpression enhances salt tolerance in Arabidopsis.

### 2.8. Detection of Stress-Resistant Physiological Indicators in A. thaliana Under Various Stress Treatments

**Figure 5:**
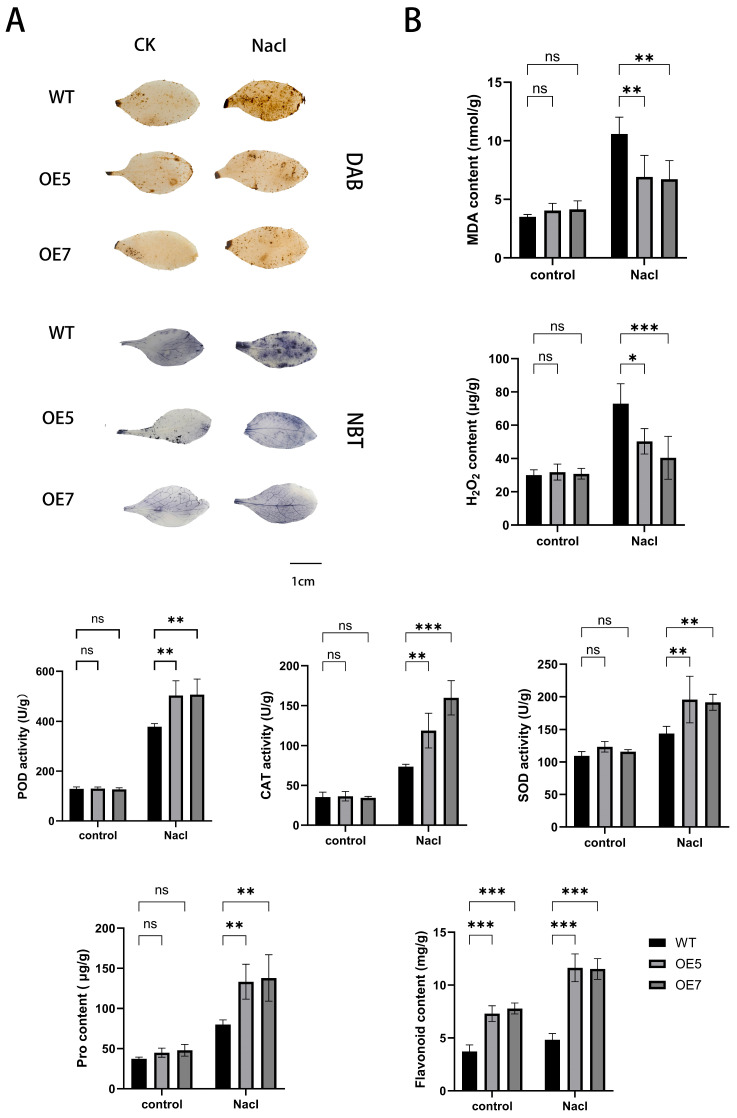
Oxidative stress responses and antioxidant enzyme activities in WT and *AcCHS5*-OE lines (OE5, OE7) under control and NaCl stress conditions. (**A**) Phenotypic visualization using DAB (brown coloration for H_2_O_2_ accumulation) and NBT (blue coloration for O_2_·^−^ detection) staining. Scale bar = 1 cm. (**B**) Biochemical quantification of MDA, H_2_O_2_ content, POD, CAT, SOD activities, Pro content, and flavonoid content. Black bars: WT; light gray: OE5; dark gray: OE7. Error bars represent mean ± SD from three biological replicates (n = 3). Statistical significance was determined using two-way ANOVA (ns, * *p* < 0.05, ** *p* < 0.01, *** *p* < 0.001).

Salt stress typically induces severe oxidative damage in plants, subsequently triggering a secondary stress-oxidative stress cascade. Under stress conditions, plants accumulate elevated levels of H_2_O_2_ and O_2_·^−^, which can destabilize lipids, proteins, and nucleic acids, thereby disrupting normal cellular metabolism [23]. Following salt stress treatment, DAB(3,3′-Diaminobenzidine) and NBT (Nitroblue tetrazolium) staining of *A. thaliana* leaves from different transgenic lines revealed no significant chromatic differences between the wild-type (WT) and *AcCHS5*-OE lines under normal cultivation conditions. However, under salt stress, the *AcCHS5*-OE lines exhibited attenuated blue (NBT) and brown (DAB) staining intensities compared to the WT lines, indicating the reduced accumulation of H_2_O_2_ and O_2_·^−^ in leaves (Figure 5A).

Physiological parameters including flavonoid content, H_2_O_2_ content, Pro content, MDA content, and antioxidant enzyme activities (SOD, CAT, and POD) were quantified in both the WT and *AcCHS5*-OE lines under salt-stressed and control conditions. Under control conditions, the *AcCHS5*-OE lines exhibited a significantly higher flavonoid content compared to the WT lines. This differential was amplified under salt stress, with the *AcCHS5*-OE lines maintaining substantially elevated flavonoid levels relative to the WT lines. Upon the high-salinity challenge, both malondialdehyde (MDA) content and H_2_O_2_ content showed significant reductions, while antioxidant enzyme activities (SOD, CAT, and POD) and Pro accumulation demonstrated marked increases in the *AcCHS5*-OE lines compared to the WT lines (Figure 5B).

### 2.9. Correlation Analysis of MYB Transcription Factors and Flavonoid Biosynthesis Genes in A. catechu 

**Figure 6:**
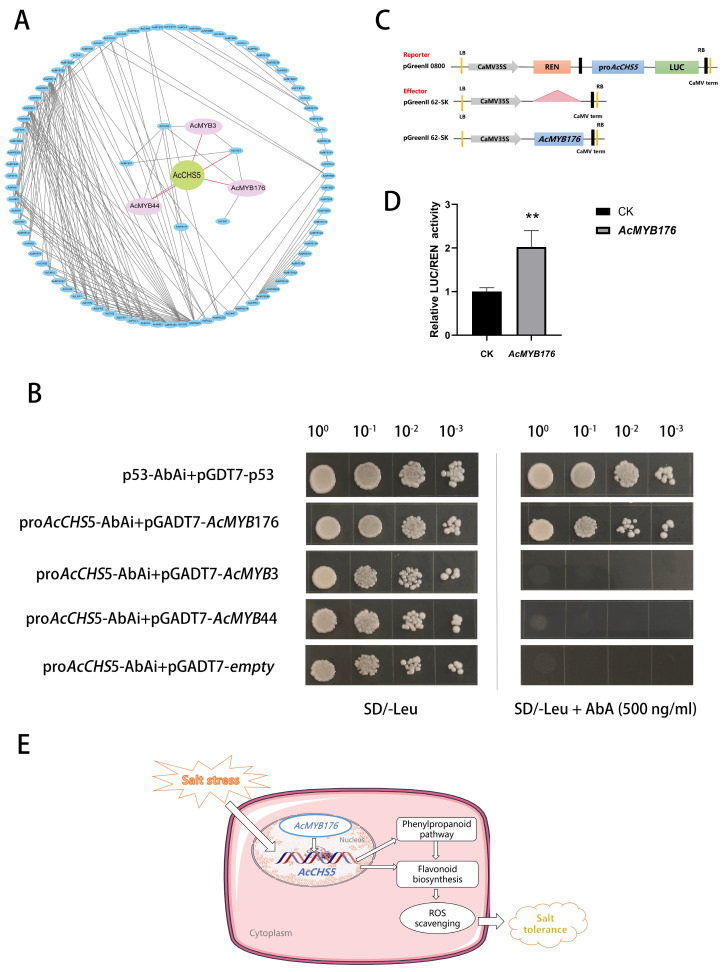
Molecular interaction and transcriptional regulation of *AcCHS5* in flavonoid biosynthesis. (**A**) Correlation analysis network of flavonoid biosynthetic genes with MYB transcription factors. Central green node: *AcCHS5*; interacting partners (pink nodes): *AcMYB3*, *AcMYB44*, *AcMYB176*. (**B**) Yeast one-hybrid (Y1H) assay validating binding of MYB TFs (*AcMYB176*, *AcMYB3*, *AcMYB44*) to the *AcCHS5* promoter (pro*AcCHS5*). Controls: p53-AbAi + pGADT7-p53 (positive), pro*AcCHS5*-AbAi + pGADT7-empty (negative). Yeast suspensions (10^0^–10^−3^ dilutions) spotted on SD/-Leu ± 500 ng/mL AbA. (**C**) Schematic diagram of the dual-luciferase reporter and effector vectors. (**D**) Dual-luciferase assay showing transcriptional activation of *AcCHS5* by *AcMYB176*. Control group: pro*AcCHS5*-pGreenII 0800-LUC + empty-pGreenII 62-SK vector. Experimental group: pro*AcCHS5*-pGreenII 0800-LUC + *AcMYB176*-pGreenII 62-SK. Error bars represent mean ± SD from three biological replicates (n = 3). Statistical significance was determined using *t*-test (** *p* < 0.01 vs. CK.). (**E**) Model of the *AcMYB176-AcCHS5* module regulating flavonoid biosynthesis in response to salt stress. Salt stress induces the expression of the flavonoid biosynthetic gene *AcCHS5* in *A. catechu*, where the transcription factor *AcMYB176* transcriptionally activates *AcCHS5* to enhance flavonoid biosynthesis, thereby scavenging ROS and ultimately regulating plant salt tolerance.

Building upon our previous identification of the *AcMYB* gene family in *A. catechu* [24]*,* this study investigated the regulatory roles of *AcMYB* transcription factors in flavonoid biosynthesis. Utilizing transcriptomic datasets and Pearson correlation coefficients (PCCs), we conducted a systematic correlation analysis between the *AcMYB* genes and flavonoid pathway genes. The top 100 positively/negatively correlated gene pairs were identified, with three MYB regulators (*AcMYB176*, *AcMYB44*, and *AcMYB3*) showing exceptionally strong positive correlations with *AcCHS5* (*AcMYB176-AcCHS5*, PCC = 0.997; *AcMYB3-AcCHS5*, PCC = 0.986; and *AcMYB44-AcCHS5*, PCC = 0.975) (Figure 6A, Table S2). Subsequent studies will focus on the functional validation of these candidate regulators.

### 2.10. Yeast One-Hybrid Assay Validation

All yeast transformants grew successfully on SD/-Leu medium, validating the efficient transformation of the pGADT7 plasmid into bait yeast strains. When subjected to selective pressure on SD/-Leu medium supplemented with 500 ng/mL Aureobasidin A (AbA), the positive control (p53-pGADT7, a validated interaction pair) and the *AcMYB176*-pGADT7 construct exhibited sustained proliferation, whereas the negative control (empty pGADT7) failed to grow (Figure 6B). This stringent interaction assay confirmed the specific binding affinity between *AcMYB176* and the *AcCHS5* promoter (pro*AcCHS5*).

### 2.11. Dual-Luciferase Assay Results Analysis

To investigate the transcriptional regulatory role of *AcMYB176* on the *AcCHS5* promoter, we employed an Agrobacterium GV3101-mediated transient expression system for genetic transformation. Recombinant constructs were introduced into the abaxial epidermis of Nicotiana benthamiana leaves through infiltration. Subsequent dual-luciferase reporter assays conducted 48 h post-transformation revealed distinct transcriptional activation patterns, quantified through the ratio of firefly luciferase (LUC) to Renilla luciferase (REN) enzymatic activities. The pro*AcCHS5* promoter was used to drive the LUC gene as the reporter, while *AcMYB176* was cloned into the pGreenII 62-SK vector as the effector (Figure 6C). The result demonstrated a statistically significant 2.02-fold elevation (*p* < 0.01) in the LUC/REN activity ratio in the *AcMYB176*-expressing tissues relative to the empty vector control (Figure 6D). This evidence substantiates the specific binding capacity of transcription factor *AcMYB176* to pro*AcCHS5* and its functional role in enhancing promoter-driven transcriptional activation.

## 3. Discussion

In this study, we conducted a genome-wide identification of the *CHS* gene family in *A. catechu* through a bioinformatics analysis and performed a functional characterization of *AcCHS5*. The MYB transcription factor *AcMYB176* was demonstrated to act as a positive regulator by binding to the promoter region of *AcCHS5*, thereby promoting flavonoid biosynthesis and enhancing salt tolerance in *A. catechu*. These findings provide molecular insights into the regulatory network underlying salt stress adaptation in *A. catechu*, offering valuable references for the molecular breeding of stress-resistant cultivars. The results hold significant implications for developing innovative cultivation models for crops in tropical coastal saline-alkali lands and advancing ecological resource utilization.

This study identified seven *CHS* genes in *A. catechu* (Table S1), contrasting with reported counts in other species: four in cucumber (*Cucumis sativus* L.) [25], seven in eggplant (*Solanum melongena* L.) [26], and fourteen in common bean (*Phaseolus vulgaris* L.) [27], highlighting interspecific variation in the *CHS* family expansion. A phylogenetic reconstruction revealed the closest evolutionary relationships were between *A. catechu* and those of *C. nucifera tall* and *C. nucifera dwarf*, suggesting functional conservation between these palm species (Figure 1A). Comparative genomic analyses further demonstrated stronger syntenic conservation between *A. catechu* and *C. nucifera dwarf* and *C. nucifera tall,* than with *A. thaliana*, *O. sativa*, or *E. guineensis*, supporting their shared ancestry within the Arecaceae family (Figure 1C). The Ka/Ks ratio, a statistical metric quantifying evolutionary rates in gene sequences, was employed to assess selective pressures by comparing the frequency of nonsynonymous substitutions (Ka) to synonymous substitutions (Ks) [28]. To elucidate evolutionary selection patterns, we calculated nonsynonymous/synonymous substitution ratios (Ka/Ks) for syntenic *CHS* gene pairs. All analyzed pairs exhibited Ka/Ks < 1, indicative of pervasive purifying selection. This evolutionary constraint likely preserves the structural and functional integrity of *CHS* genes by eliminating deleterious nonsynonymous mutations during speciation (Table 1).

Cis-regulatory elements serve as key determinants in transcriptional control. Specific binding sites within promoter regions, particularly their interactions with transcription factors, critically determine the precision of transcriptional control [29]. Characterizing cis-regulatory elements within the *AcCHS* gene family revealed that their upstream promoter regions harbor multiple stress-responsive *cis*-regulatory motifs, including light-responsive elements (e.g., G-box), stress-responsive elements (e.g., MYB), hormone-responsive elements (e.g., AREB), and plant development elements (e.g., CAT-box) (Figure 2A,B). Similarly, previous studies have identified critical cis-acting elements in the *CHS* gene promoter regions, including AREB (ABA-responsive element binding) elements [30], G-box elements [31], and MYB recognition sites [32]. Notably, research has demonstrated that MYB transcription factors regulate *CHS* gene expression by binding to these MYB-specific cis-elements within the promoter region, thereby modulating plant salt tolerance through flavonoid biosynthesis pathways [33].

Salt stress exerts profound inhibitory effects on plant growth and development, typically manifested through growth retardation, dwarfism, and leaf chlorosis [34]. In this study, the *AcCHS5*-OE lines demonstrated superior phenotypic performance under salt stress compared to the WT plants, exhibiting significantly enhanced root elongation, increased biomass accumulation, and attenuated leaf chlorosis (Figure 4B–E). The observed morphological advantages suggest that *AcCHS5* overexpression may improve water uptake efficiency and promote root system development, thereby mitigating salt-induced growth suppression. 

Plants deploy sophisticated regulatory networks to counteract salt stress, including the activation of antioxidant enzymes and accumulation of antioxidants [35]. Our results corroborate previous studies in *Iris halophila* Pall., in which *CHS* gene overexpression enhanced salt tolerance through reduced membrane lipid peroxidation and increased flavonoid compound biosynthesis [36]. The current study extends these observations by demonstrating that *AcCHS5-OE* lines exhibit significantly lower oxidative damage markers (H_2_O_2_ and MDA content) alongside elevated antioxidase activities (SOD, CAT, and POD) and Pro accumulation (Figure 5B). This coordinated enhancement of the ROS scavenging system effectively maintains cellular redox homeostasis, reducing oxidative injury at both subcellular and whole-plant levels. Specifically, The SOD-CAT-POD enzymatic cascade synergistically interacts with the osmoprotectant (Pro) to regulate redox homeostasis via the sequential conversion of O_2_•^−^ to H_2_O_2_ and subsequent decomposition to water (H_2_O), accompanied by the concurrent scavenging of hydroxyl radicals (·OH). This integrated system operates in subcellular compartments: in chloroplasts, it mitigates photosystem II (PSII) photoinhibition to maintain photosynthetic efficiency, whereas in mitochondria, it preserves electron transport chain integrity to ensure ATP production. By coordinating ROS detoxification across cellular compartments, this mechanism attenuates salt-induced oxidative damage at both cellular and whole-plant levels, thereby conferring salt tolerance to plants [37].

The phenolic hydroxyl groups in flavonoid compounds significantly enhance their reductive capacity, conferring both radical-scavenging potential and antioxidant functionality [38]. Furthermore, flavonoids can induce the expression of multiple enzymatic systems in various cell types, including SOD, CAT, POD heme oxygenase-1 (HO-1), and glutathione peroxidase (GPX) [39]. In this study, the *AcCHS5*-OE lines exhibited substantially higher flavonoid accumulation compared to the WT plants under salt stress, corroborating the role of *AcCHS5* in enhancing flavonoid biosynthesis and consequently improving salt tolerance in *A. catechu* (Figure 5B). These findings align with studies in tea plants (*Camellia sinensis* (L.) Kuntze), in which three *CsCHS* genes were identified and demonstrated to restore flavonoid synthesis in *A. thaliana CHS* mutants, with *CsCHS*-transgenic tobacco showing an elevated flavonoid content relative to wild-type controls [40]. Collectively, these studies demonstrate that *CHS* genes enhance plant oxidative stress tolerance by promoting flavonoid biosynthesis and regulating antioxidant enzyme systems, thereby maintaining ROS homeostasis.

Substantial evidence indicates that MYB transcription factors play pivotal roles in regulating flavonoid biosynthesis pathways. In *Lilium lancifolium* Thunb., the *LlMYB3* transcription factor binds to MYB recognition motifs within the *LlCHS* promoter, functioning as an upstream regulator to enhance salinity tolerance in overexpression lines [41]. Similarly, *A. thaliana* studies have identified *MYB111* as a positive regulator that interacts with cis-elements in the *AtCHS* promoter, promoting transcriptional activation and flavonoid accumulation under salt stress [42]. In this study, a correlation analysis of *AcMYB* genes and flavonoid biosynthesis-related genes predicted *AcMYB176*, *AcMYB3*, and *AcMYB44* as potential regulatory candidates (Figure 6A). Our experimental validation confirmed that *AcMYB176* directly interacts with the *AcCHS5* promoter via yeast one-hybrid assays (Figure 6B). Furthermore, dual-luciferase reporter assays demonstrated that *AcMYB176* acts as a transcriptional activator of *AcCHS5*, establishing its role in modulating flavonoid-mediated salt adaptation mechanisms (Figure 6C).

## 4. Materials and Methods

### 4.1. Phylogenetic Analysis of the CHS Gene Family in A. catechu 

The genome of *A. catechu* was retrieved from the NCBI database (GenBank accession: JAHSVC000000000; BioSample: SAMN19591864). Genomic sequences of *A. thaliana* and *O. sativa* were obtained from Ensembl Plants database (https://plants.ensembl.org) (accessed on 10 November 2024), while palm species (*C. nucifera tall*, *C. nucifera dwarf*, *P. dactylifera*, and *E. guineensis*) data were sourced from the Arecaceae Genome Database (https://arecaceae-gdb.org) (accessed on 10 November 2024). The amino acid sequences of *CHS* gene family members in *A. thaliana* were downloaded from TAIR (https://www.arabidopsis.org) (accessed on 10 November 2024). *CHS* homologs in *A. catechu* were identified via BLASTP against *A. thaliana* references (E-value < 1 × 10^−^^5^, identity > 40%), followed by domain validation using HMMER (version 3.0) with the Pfam *CHS* model (PF00195; E-value < 0.01). CHS protein sequences from seven species were aligned in TBtools (version 2.1025), and a neighbor-joining tree was constructed in MEGA11 (Poisson model, 1000 bootstraps). Final visualization was performed using iTOL (https://itol.embl.de) (accessed on 20 November 2024).

### 4.2. Inter- and Intra-Species Collinearity Analysis of the CHS Gene Family in A. catechu

The TBtools software was launched to extract chromosomal localization data of *CHS* genes using the Fasta Stats tool. Collinearity analysis was then conducted through the One-Step MCScanX module with default parameters, and the output files were saved for downstream analyses. The collinearity relationships were visualized at high resolution using the Advanced Circos toolkit. For evolutionary rate estimation, syntenic gene pairs were first identified with the Simple Ka/Ks Calculator, followed by Ka and Ks substitution rates. These quantitative metrics provide critical insights into the evolutionary constraints acting on *CHS* genes.

### 4.3. Cis-Acting Element Analysis of the CHS Gene Family in A. catechu

The TBtools software was initialized to extract 2000 bp upstream regions of coding sequences (CDS) from *A. catechu CHS* genes using the GFF3 Sequence Extraction module. The processed sequences were then submitted to the PlantCARE database (https://bioinformatics.psb.ugent.be/webtools/plantcare/html/) (accessed on 6 December 2024) for comprehensive prediction of *cis*-acting regulatory elements. The resulting data were systematically organized in Microsoft Excel and subsequently visualized through GraphPad Prism (version 9.0) and TBtools to elucidate spatial distribution patterns of regulatory elements across the *CHS* gene family.

### 4.4. Expression Pattern Analysis of CHS Genes in A. catechu

*A. catechu* cv. ‘ReYan No.1’ samples used in this study were naturally grown at the Danzhou Campus of Hainan University. Fresh tissues including leaves, flowers (male and female), pericarps, and endosperms were collected, flash-frozen in liquid nitrogen, and stored at −80 °C. Total RNA was isolated using the Plant RNA Extraction Kit (Accurate, Beijing, China) and subsequently reverse-transcribed into cDNA with the FastKing cDNA Synthesis Kit (Vazyme, Nanjing, China), which served as the template for subsequent quantitative analyses. Target-specific primers for qPCR were developed using Premier (version 5.0) (Table S3). Expression profiles of *CHS* genes were systematically analyzed in leaves, male flowers, female flowers, pericarps, and endosperms through qPCR with three biological replicates per sample.

Uniformly developed *A. catechu* seedlings were transplanted into 12 × 12 cm plastic pots filled with perlite substrate and maintained in a plant growth facility with precisely controlled conditions (14 h photoperiod and 10 h dark cycle). Plants were irrigated every 4 days. After 14 days of acclimatization, seedlings were divided into two groups: (1) a control group (normal irrigation); and (2) an experimental group subjected to salt stress via irrigation with 200 mmol·L^−^^1^ NaCl solution. Foliar tissues of NaCl-stressed and untreated plants were harvested at 1, 3, 7, and 14 days after treatment, snap-frozen using liquid nitrogen, and maintained at −80 °C for preservation. Expression levels of *CHS* genes in leaves were quantified by qPCR to compare transcriptional responses between salt-stressed and untreated plants across time points, with three biological replicates per group.

### 4.5. Construction of AcCHS5 Overexpression Vector and Generation of Transgenic A. thaliana

Based on the coding sequence (CDS) of *AcCHS5* and the multiple cloning sites of the pCAMBIA-1300 vector, restriction enzymes SpeI and XbaI were selected for gene cloning. Gene-specific primers were designed for molecular cloning (Table S4). The target fragment was PCR-amplified from *A. Catechu* cDNA with Vazyme’s high-fidelity polymerase, followed by purification via the manufacturer’s gel extraction kit (Vazyme). The purified fragment was ligated into the Blunt-Zero cloning vector (Vazyme) and transformed into *Escherichia coli* DH5α competent cells, followed by incubation at 37 °C. Positive clones were screened by colony PCR and sent to Sangon Biotech (Shanghai, China) for Sanger sequencing. Sequence-verified T-vectors were subjected to double digestion with SpeI/XbaI, and the excised fragment was gel-purified. The digested fragment was ligated into the linearized pCAMBIA-1300 expression vector, followed by restriction digestion verification to confirm successful insertion. The recombinant pCAMBIA-1300-*AcCHS5* construct was introduced into *Agrobacterium tumefaciens* strain GV3101. *A. thaliana* (Col-0) inflorescences were dip-inoculated with the *Agrobacterium* suspension harboring the target construct. Seeds were harvested at maturity and surface-sterilized before sowing on ½ MS medium containing 30 mg/L hygromycin. Transgenic plants were selected over three generations (T3). Homozygous lines with high *AcCHS5* expression levels, as confirmed by qRT-PCR, were retained for subsequent experiments.

### 4.6. Phenotypic Observation of A. thaliana Under Salt Stress Treatment

WT (Col-0) and T3-generation *AcCHS5-OE Arabidopsis* seeds underwent surface sterilization via sequential immersion in 75% ethanol (8 min) and 5% sodium hypochlorite (5 min), followed by triple rinsing with sterile distilled water. Seeds were sown on ½ MS agar medium and cultured in a growth chamber under controlled conditions (22 °C, 16 h photoperiod, and light intensity of 120 μmol·m^−^^2^·s^−^^1^). Stress treatments were applied to both seedlings and adult plants, with non-stressed wild-type and two transgenic lines serving as controls. All treatments included three biological replicates. Seedling stress treatment: ½ MS agar plates containing 200 mmol·L^−^^1^ NaCl (Sigma-Aldrich, St. Louis, MO, USA) were prepared for salt stress induction. Seven-day-old *A. thaliana* seedlings were transferred to the stress medium and cultured for 7 days. ImageJ (version 2.9.0) was employed to analyze root length parameters. Adult plant stress treatment: A growth substrate was prepared by mixing Danish Pindstrup peat and vermiculite at a 3:1 ratio (*v/v*). Twenty-one-day-old plants were irrigated with 200 mmol·L^−^^1^ NaCl solution for 14 days to induce salt stress.

### 4.7. Detection of Stress-Resistant Physiological Indices in A. thaliana Under Salt Stress

Whole plants were collected for physiological analysis after salt stress treatment. Pooled samples consisting of 3–6 wild-type (*A. thaliana* Col-0) plants and three plants per transgenic line were used for biochemical assays. The following stress-related indices were quantified using commercial assay kits (Solarbio, Beijing, China): flavonoid content, Pro content, MDA content, H_2_O_2_ accumulation, CAT activity, SOD activity, and POD activity.

### 4.8. NBT Staining and DAB Staining

NBT (Nitroblue tetrazolium) staining was employed to detect O_2_·^−^ accumulation, while DAB (3,3′-Diaminobenzidine) staining was used to visualize H_2_O_2_ distribution. The seventh leaf from 28-day-old *A. thaliana* seedlings were immersed in NBT and DAB staining solutions (Solarbio, Beijing, China), respectively, for 3–6 h. Subsequently, the stained leaves were decolorized in absolute ethanol for 8 h to remove residual pigments. After thorough rinsing to eliminate nonspecific staining, the samples were photographed under standardized imaging conditions.

### 4.9. Correlation Analysis of MYB Transcription Factors and Flavonoid Biosynthesis Genes in A. catechu 

Utilizing transcriptomic data from *A. catechu*, we first identified transcript sequences of the *AcMYB* transcription factor family members and genes associated with the flavonoid biosynthetic pathway through genome annotation screening. Pearson correlation analysis was conducted using the stats package in R (version 4.2.1), with *AcMYB* genes designated as the query gene set and flavonoid synthesis-related genes as the target gene set for pairwise correlation calculation. A significance threshold of *p* < 0.05 (adjusted via the Benjamini–Hochberg method) was applied to select the top 100 high-confidence interaction pairs exhibiting both positive and negative correlations. Network visualization was subsequently generated using Cytoscape software (version 3.9.1).

### 4.10. Yeast One-Hybrid Vector Construction

The 2000 bp upstream sequence of *AcCHS5* was extracted using TBtools and PCR-amplified using genomic DNA from *A. catechu* as the template (Table S4). Predicted transcription factor sequences were cloned from *A. catechu* cDNA (Table S4). The pro*AcCHS5* fragment was inserted into the pAbAi vector to construct the bait vector pro*AcCHS5*-AbAi. Three predicted transcription factors (*AcMYB176*, *AcMYB44*, and *AcMYB3*) were ligated into the pGADT7 vector, generating prey vectors for subsequent interaction assays.

### 4.11. Screening of Bait Yeast Strain Sensitivity to AbA

Yeast colonies identified as positive on SD/-Ura plates were transferred to SD/-Ura liquid medium and grown for two days at 28 °C in a shaking incubator (200 rpm). The cells were resuspended in 0.9% NaCl solution and diluted to an OD_600_ of 0.002 (equivalent to 2000 cells per 100 μL suspension). Aliquots (100 μL) of the diluted culture were plated onto SD/-Ura agar plates containing varying concentrations of AbA: 100, 200, 300, 400, and 500 ng/mL. Following 3-day incubation at 28 °C, the minimum inhibitory concentration (MIC) of AbA required to suppress bait strain growth was determined based on colony viability assessment.

### 4.12. Yeast One-Hybrid Interaction Assay

The constructed plasmid *AcMYB*-pGADT7 was co-transformed into pro*AcCHS5*-AbAi-containing yeast competent cells using the polyethylene glycol/lithium acetate (PEG/LiAc) method. Cell suspensions were adjusted to OD_600_ values of 0.2, 0.02, 0.002, and 0.0002 (ten-fold serial dilutions) and spotted onto both SD/-Leu medium and SD/-Leu medium supplemented with the MIC of AbA. Plates were transferred to a 30 °C incubator for a period of 3–5 days. Reporter gene activation, evidenced by yeast growth on selective media, occurred only if the target transcription factor (*AcMYB*) interacted with the pro*AcCHS5* promoter sequence. Observable colony development under AbA selection confirmed protein–DNA interaction.

### 4.13. Dual-Luciferase Reporter Assay (LUC)

The recombinant vectors pro*AcCHS5*-pGreenII 0800-LUC and pGreenII 62-SK-*AcMYB176* were constructed and independently transformed into GV3101 competent cells. Mature leaves of *N. benthamiana* plants at the 4-week growth stage were harvested for Agrobacterium-mediated transient expression assays. The bacterial cultures, following resuspension, were blended in a 1:1 volumetric ratio prior to syringe-mediated delivery into the abaxial leaf epidermis of tobacco plants. The following combinations were used: control group of pro*AcCHS5*-pGreenII 0800-LUC +empty pGreenII 62-SK vector; and experimental group of pro*AcCHS5*-pGreenII 0800-LUC + *AcMYB176*-pGreenII 62-SK. Infiltrated plants were incubated in the dark for 24 h followed by cultivation for 48 h photoperiod. Dual-luciferase activity was quantified using the Dual-Luciferase^®^ Reporter Assay Kit (Puyintech, Qingdao, China). The relative transcriptional activity was calculated as the ratio of firefly luciferase (LUC) to Renilla luciferase (REN) luminescence signals. Three biological replicates were performed for each experimental condition.

### 4.14. Data Analysis

The experimental data were initially organized using Microsoft Excel, followed by statistical significance analysis performed with SPSS (version 21.0). Based on the experimental design, independent sample *t*-test and two-way analysis of variance (two-way ANOVA) were selected for hypothesis testing. Visualization of data and statistical significance annotations were subsequently conducted using GraphPad Prism (version 9.0). The asterisk (*) denotes a *p*-value < 0.05, (**) indicates *p* < 0.01, (***) represents *p* < 0.001, and ns means not significant. 

## 5. Conclusions

Our findings demonstrate that *AcMYB176* directly binds to the promoter of *AcCHS5*, positively activating and enhancing its transcriptional activity. This regulatory interaction promotes flavonoid biosynthesis, scavenges reactive ROS, and ultimately improves salt tolerance in *A. catechu.* Additionally, we propose a potential response model (Figure 6E). Elucidating the molecular mechanisms underlying salt tolerance and the regulatory network of flavonoid metabolism in *A. catechu* not only paves the way for genetic breeding improvements through molecular approaches, but also provides both the theoretical foundation and technical support for high-quality molecular breeding programs.

## Figures and Tables

**Table 1:** Non-synonymous (Ka) and synonymous (Ks) substitution rates with Ka/Ks ratios across species comparisons.

Species	Seq1	Seq2	Ka	Ks	Ka/Ks
Ac vs. Az	AC07G001270.1	AZ01G0004910.1	0.019008	0.34062	0.055803
Ac vs. Az	AC07G001270.1	AZ07G0156950.1	0.060103	1.375058	0.043709
Ac vs. Az	AC07G001270.1	AZ12G0221420.1	0.012157	0.249037	0.048816
Ac vs. Az	AC10G052970.1	AZ01G0004910.1	0.053033	0.889335	0.059633
Ac vs. Az	AC10G052970.1	AZ07G0156950.1	0.011962	0.180102	0.066416
Ac vs. Az	AC11G020520.1	AZ01G0004910.1	0.077037	0.263941	0.291871
Ac vs. Gz	AC07G001270.1	GZ01G0005140.1	0.019008	0.34062	0.055803
Ac vs. Gz	AC07G001270.1	GZ07G0167730.1	0.069142	1.231382	0.05615
Ac vs. Gz	AC07G001270.1	GZ12G0234890.1	0.012157	0.249037	0.048816
Ac vs. Gz	AC10G052970.1	GZ01G0005140.1	0.053033	0.889335	0.059633
Ac vs. Gz	AC10G052970.1	GZ07G0167730.1	0.014559	0.191921	0.07586
Ac vs. Gz	AC10G052970.1	GZ12G0234890.1	0.057815	1.070942	0.053985
Ac vs. Gz	AC11G020520.1	GZ01G0005140.1	0.077037	0.263941	0.291871
Ac vs. At	AC07G001270.1	AT5G13930.1	0.102025	2.071949	0.049241
Ac vs. Os	AC07G001270.1	Os11t0530600-01	0.102015	0.697797	0.146195
Ac vs. Eg	AC07G001270.1	XM_010931189.3	0.015307	0.211388	0.07241

Note: Species abbreviations: Ac (*A. catechu*), Az (*C. nucifera dwarf*), Gz (*C. nucifera tall*), At (*A. thaliana*), Os (*O. sativa*), Eg (*E. guineensis*). Seq1 and Seq2 denote compared gene pairs.

## Data Availability

The data generated and analyzed in this study are available upon request.

## Supplementary Materials

The following supporting information can be downloaded at: https://www.mdpi.com/article/10.3390/ijms26073216/s1.
